# Defining the dose‐volume criteria for laryngeal sparing in locally advanced oropharyngeal cancer utilizing split‐field IMRT, whole‐field IMRT and VMAT

**DOI:** 10.1002/acm2.13009

**Published:** 2020-12-05

**Authors:** Christopher Wilke, Vinita Takiar, He Wang, Amy C. Moreno, Shih‐Ming Samuel Tung, Sean R. Quinlan‐Davidson, Adam S. Garden, David I. Rosenthal, Clifton D. Fuller, Gary B. Gunn, Jay P. Reddy, William H. Morrison, Congjun Wang, George Zhao, Katherine A. Hutcheson, Jack Phan

**Affiliations:** ^1^ Department of Radiation Oncology The University of Texas MD Anderson Cancer Center Houston TX USA; ^2^ Department of Radiation Oncology University of Cincinnati Cincinnati OH USA; ^3^ Department of Radiation Physics The University of Texas MD Anderson Cancer Center Houston TX USA; ^4^ Department of Head and Neck Surgery The University of Texas MD Anderson Cancer Center Houston TX USA

**Keywords:** dosimetry, IMRT, Larynx, oropharynx, split‐field, VMAT

## Abstract

**Purpose:**

To determine the optimal dose‐volume constraint for laryngeal sparing using three commonly employed intensity modulated radiation therapy (IMRT) approaches in patients with oropharyngeal cancer treated to the bilateral neck.

**Materials and methods:**

Thirty patients with stage II‐IVA oropharynx cancers received definitive radiotherapy with split‐field IMRT (SF‐IMRT) to the bilateral neck between 2008 and 2013. Each case was re‐planned using whole‐field IMRT (WF‐IMRT) and volumetric modulated arc therapy (VMAT) and plan quality metrics and dose to laryngeal structures was evaluated. Two larynx volumes were defined and compared on the current study: the Radiation Therapy Oncology Group (RTOG) larynx as defined per the RTOG 1016 protocol and the MDACC larynx defined as the components of the larynx bounded by the superior and inferior extent of the thyroid cartilage.

**Results:**

Target coverage, conformity, and heterogeneity indices were similar in all techniques. The RTOG larynx mean dose was lower with WF‐IMRT than SF‐IMRT (22.1 vs 25.8 Gy; *P* < 0.01). The MDACC larynx mean dose was 17.5 Gy ± 5.4 Gy with no differences between the 3 techniques. WF‐IMRT and VMAT plans were associated with lower mean doses to the supraglottic larynx (42.1 vs 41.2 vs 54.8 Gy; *P* < 0.01) and esophagus (18.1 vs 18.2 vs 36 Gy; *P* < 0.01).

**Conclusions:**

Modern whole field techniques can provide effective laryngeal sparing in patients receiving radiotherapy to the bilateral neck for advanced oropharyngeal cancers.

**Summary:**

We evaluated laryngeal dose in patients with locally advanced oropharyngeal cancer treated to the bilateral neck using split‐field IMRT (SF‐IMRT), whole‐field IMRT (WF‐IMRT) and volumetric arc therapy (VMAT). All three techniques provided good sparing of laryngeal structures and were able to achieve a mean larynx dose < 33 Gy. There were no significant differences in dose to target structures or non‐laryngeal organs at risk among techniques.

## 1 INTRODUCTION

Head and neck cancer mortality in the United States has steadily fallen over the past several decades in part because of the increasing incidence of human‐papilloma virus (HPV) associated oropharyngeal squamous carcinoma (OPSCC). As more patients are cured of their disease, the focus of curative radiotherapy has shifted towards minimizing treatment‐related toxicities.^1^, ^2^, ^3^ Dysphagia is one of the more commonly cited long‐term side effects associated with head and neck radiotherapy and has been shown to significantly decrease quality of life (QOL) metrics following treatment.^4^, ^5^ It is of considerable interest, therefore, to minimize dose to the larynx and laryngeal substructures while maintaining optimized dose delivery to the tumor and at risk sites.

Historically, radiotherapy for OPSCC was delivered with a three‐field approach, utilizing a parallel‐opposed beam arrangement to treat the primary tumor and lymph nodes above the thyroid cartilage. These beams were matched to a low anterior neck (LAN) field to address the draining lymphatics of the mid‐low neck and a laryngeal block was included to shield the larynx.^6^, ^7^ Although intensity modulated radiation therapy (IMRT) has since emerged as the preferred approach for the treatment of head and neck malignancies owing particularly to improved parotid sparing,^8^, ^9^ the effectiveness by which whole‐field IMRT (WF‐IMRT), which encompasses the entire target volume and low neck in a single plan, can protect the larynx compared to a larynx block LAN field remains controversial. The split‐field IMRT (SF‐IMRT) technique was employed as an effort to minimize excess dose to the larynx by matching the IMRT field above the glottis to a LAN field with larynx block.^10^, ^11^, ^12^ However, potential drawbacks with SF‐IMRT are longer treatment times, dose uncertainties at the match line and the technical challenges of incorporating mid‐neck and appositional electron boost fields in patients with gross disease at or below the match line.

In our previous study, we compared SF‐IMRT to whole‐field‐IMRT (WF‐IMRT) and found the former to provide significantly better laryngeal sparing (mean laryngeal dose: 18.7 vs 47 Gy).^13^ There have been a number of notable technological advances since our initial study was performed. Volumetric modulated arc therapy (VMAT) represents a new IMRT technology that offers improved treatment efficiency and dose conformity, but it can be difficult to match to LAN fields.^14^, ^15^ Improved diagnostic imaging accuracy in CT‐based target and organ at risk (OAR) delineation together with image‐guided radiation therapy (IGRT) have additionally allowed smaller treatment margins to be used. Whether these improvements can improve larynx sparing compared to SF‐IMRT in the treatment of OPSCC remains unclear.

Interpretation of the dose‐volume relation for laryngeal dysfunction and identifying the *optimal* dose‐volume relationship for laryngeal dysfunction is further complicated by differences in the 3D larynx volume definition among clinical studies and in clinical practice. These differences among definitions principally involve the extent of the inclusion of structures of the supraglottic larynx. Here, we revisit larynx sparing using modern IMRT techniques in a larger and more homogeneous cohort of only oropharyngeal cancers. In particular, because of the growing acceptance of VMAT leading to a greater use of whole field techniques, we wanted to reinvestigate if this technique was compromising larynx sparing as we hypothesized over a decade ago. To further facilitate standardization of the laryngeal dose, we evaluated two common definitions of laryngeal volume in patients with locally advanced OPSCC.

## 2 MATERIALS AND METHODS

### 2.A Patients

We evaluated patients with OPSCC treated with definitive radiotherapy using SF‐IMRT at MD Anderson Cancer Center (MDACC) from 2008 to 2014. Those with the presence of bilateral or bulky nodal disease (≥6 cm), primary tumors extending to the larynx, or the absence of complete dosimetric planning data were excluded. In total, 70 patients were identified of which 30 patients were treated to the bilateral neck and included in this analysis. The study was approved by the MDACC institutional review board.

### 2.B Delineation of laryngeal and OARs volumes

**FIG. 1:**
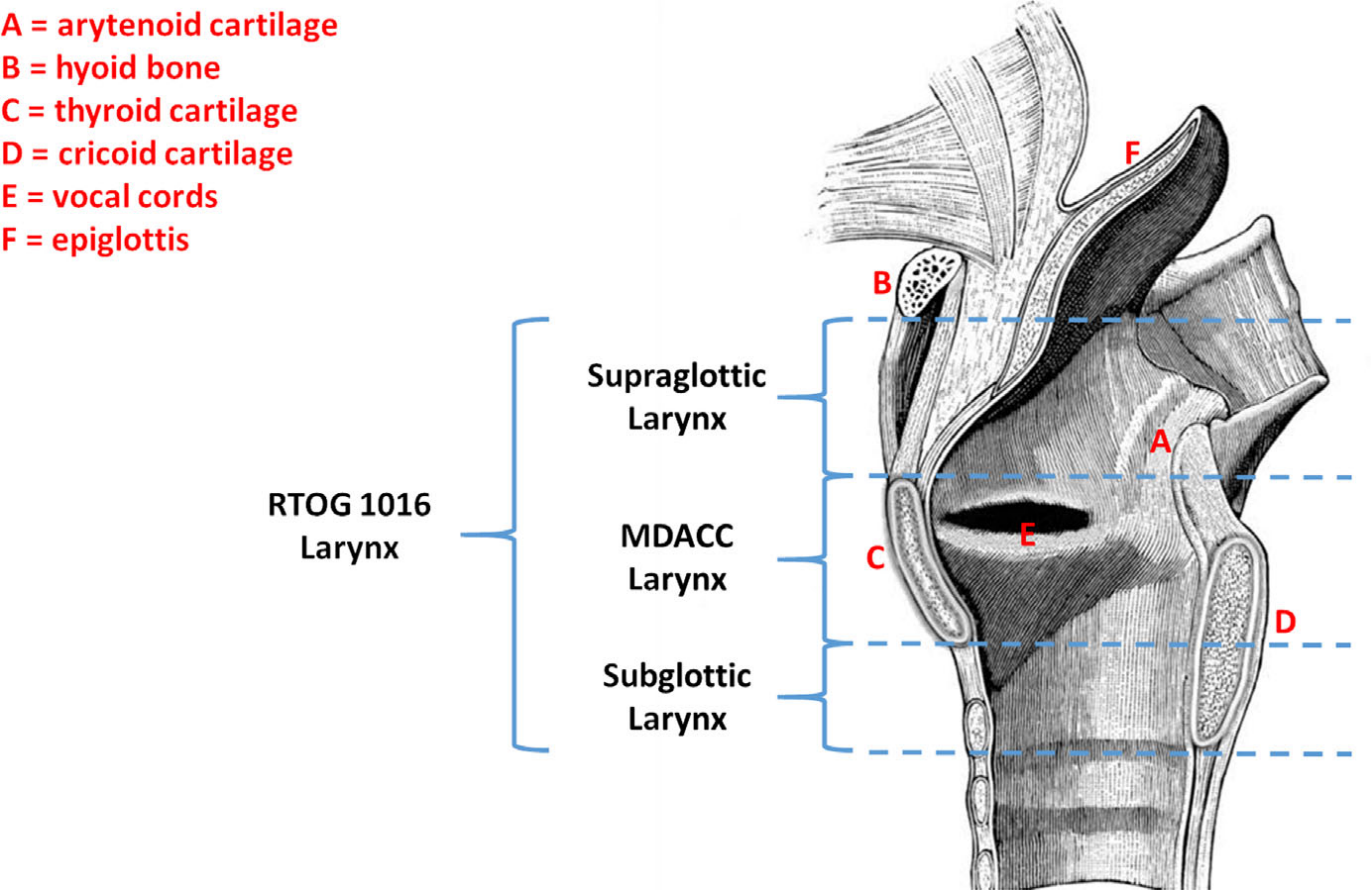
Superior and inferior borders for the laryngeal structures as defined in this study.

The larynx (MDACC larynx) was defined per our prior publication as bounded by the superior and inferior aspects of the thyroid cartilage.^13^ We defined a second larynx volume based on the Radiation Therapy Oncology Group 1016 guidelines (RTOG larynx). This volume extended from the inferior aspect of the hyoid bone to the cricoid cartilage with inclusion of the infrahyoid but not suprahyoid epiglottis. We defined the supraglottic and subglottic larynx as the volumes of the RTOG larynx extending superiorly and inferiorly, respectively, beyond the borders of the MDACC larynx. A diagram depicting the superior and inferior borders of these structures is shown in Fig. 1. Additional OARs included the spinal cord, brainstem, esophagus, arytenoids and cricopharyngeus muscle which was defined as posterior to the cricoid cartilage and extending inferiorly to the upper cervical esophagus delineated as the inferior aspect of the cricoid cartilage.

### 2.C Radiotherapy planning procedures

Contours underwent institutional quality assurance peer‐review by radiation oncologists specializing in the treatment of head and neck malignancies.^16^ The radiation treatment plans were generated using the Pinnacle treatment planning software (Philips Healthcare, Andover, MA). For the SF‐IMRT technique, the isocenter was placed at time of CT simulation and was typically located 1–1.5 cm superior to the level of the arytenoids. IMRT to a dose of 66 Gy in 30 fractions or 70 Gy in 33 fractions was used to treat the primary tumor and upper neck above the isocenter. The mid‐low neck were treated with a matched larynx block LAN field to 50 Gy in 25 fractions, with a full midline block to shield the spinal cord added after 40 Gy in 20 fractions. Dependent upon patient anatomy and physician preference, a daily PA supplement was added below the isocenter to ensure the mid‐neck nodal regions received at least 2 Gy per fraction. Involved nodal stations below the match line were boosted with 3D photon beams to 60 Gy in 30 fractions, and gross nodal disease was boosted to 66–70 Gy utilizing appositional electrons beams. The dose‐fractionation for WF‐IMRT or VMAT replans were the same as SF‐IMRT (66 Gy in 30 fractions or 70 Gy in 33 fractions) for gross primary and nodes above the match point. The simultaneous integrated boost technique with differential dose rates was applied below the match line as follows: 60 Gy in 30 fractions (or 63 Gy in 33 fractions) to involved nodal level(s) and 54 Gy in 30 fraction (or 57 Gy in 33 fractions) to lower‐risk subclinical sites.

**FIG. 2:**
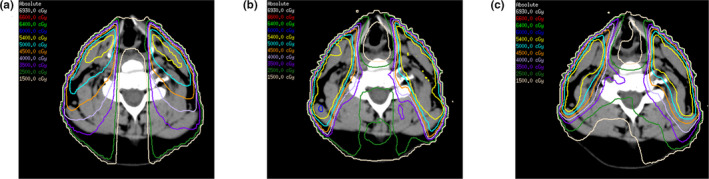
Representative isodose distributions from a single patient using (a) split‐field intensity modulated radiation therapy, (b) whole‐field intensity modulated radiation therapy and (c) volumetric arc therapy.

The planning goals for WF‐IMRT and VMAT were the same as SF‐IMRT, which were to achieve > 98% coverage for clinical target volumes (CTVs) while meeting or exceeding OAR dose constraints of SF‐IMRT plans. Additional constraints added to WF‐IMRT and VMAT during plan optimization included the larynx, esophagus and cricopharyngeus. The goal was to limit the dose to these structures to as low as possible without compromising tumor coverage as dictated by the initial SF‐IMRT planning directives. Representative isodose distributions at the laryngeal level for the SF‐IMRT, WF‐IMRT, and VMAT procedures are shown in Fig. 2.

### 2.D Statistical analysis

SF‐IMRT, WF‐IMRT, and VMAT plans were compared with regards to target coverage, OAR dose, total delivery time, monitor units required to deliver each plan, as well as the Paddick conformity index and the heterogeneity index (percentage of one standard deviation to the mean dose) of the primary targets. The Tukey‐Kramer method was used to identify significant differences between treatment methods with *P* < 0.05 considered statistically significant. All analyses were performed using the JMP Pro 12 software package (SAS, Cary, NC).

## 3 RESULTS

### 3.A Patient and treatment characteristics

Patient and treatment characteristics are shown in Table 1. The 30 OPSCC patients included in this study were comprised of 12 tonsil tumors and 18 tongue base tumors. The vast majority were T1‐T2 (28 of 30) and approximately half (14 of 30 patients) were N2b. A total of 14 patients had positive mid‐neck nodes (level III). Seventeen primary tumors received a prescribed dose of 70 Gy in 33 fractions while the remaining 13 received 66 Gy in 30 fractions. Four of the 30 patients received electron boosts as part of the SF‐IMRT treatment. In all four of these cases, the boost region was located posterior to the larynx with minimal contribution to the laryngeal dose.

**TABLE 1:** Patient characteristics.

Characteristic	Value	N
Sex	Male	28
	Female	2
Site	Tonsil	12
	Base of tongue	18
T‐stage	1	9
	2	19
	3	1
	4	1
N‐stage	0	4
	1	8
	2a	4
	2b	14
	2c	0
Group stage	I	0
	II	3
	III	8
	IVA	19
Prescribed dose	66 Gy	13
	70 Gy	17
Level 3 disease	Yes	14
	No	16

### 3.B Larynx and laryngeal substructure dose

Laryngeal/critical OAR doses and dosimetric indices are shown in Table 2. The mean dose to the MDACC larynx was lower than the RTOG larynx across all techniques. The MDACC and RTOG cumulative mean laryngeal doses were 18.1 and 25.8 Gy, respectively, for SF‐IMRT; 16.7 and 22.1 Gy, respectively for WF‐IMRT; and 16.5 and 23.0 Gy, respectively, for VMAT. In 29 of the 30 cases (97%), SF‐IMRT, WF‐IMRT, and VMAT achieved mean doses < 28 Gy for the MDACC larynx and <33 Gy for the RTOG larynx.. The lone exception was a patient with a stage T3 N1 tongue base cancer treated to 70 Gy. Review of the target structures showed the high‐dose contour extending to the vallecula and lingual epiglottis. Notably, a mean MDACC larynx dose <19 Gy was achievable in 22 of 30 (73%) SF‐IMRT cases, 21 of 30 (70%) WF‐IMRT cases and 20 of 30 (67%) VMAT cases. Tumor staging or location of primary site (tonsil vs. tongue base) did not influence the larynx dose.

**TABLE 2:** Comparison of treatment techniques.

	Split field	WF‐IMRT	VMAT
Supraglottic larynx	54.8 ± 10.8	42.1** ± 13.0	41.2** ± 12.0
Subglottic larynx	21.3 ± 8.3	19.2 ± 4.7	20.4 ± 4.4
MDACC larynx	17.8 ± 5.6	16.9 ± 4.4	18.1 ± 3.9
RTOG larynx	25.8 ± 5.1	22.1** ± 4.6	23 ± 4.4
Cricopharyngeus	13.7 ± 3.8	16* ± 3.2	17.8** ± 2.9
Arytenoids	11.9 ± 6.4	13.4 ± 4.8	14.9 ± 4.5
Esophagus	36 ± 4.8	18.1** ± 5.4	18.2** ± 5.3
Cord (mean)	24.5 ± 2.5	21.9** ± 3.5	21.2** ± 3.2
Cord (max)	39.3 ± 2.5	35.5** ± 3.7	35.2** ± 3.2
Brainstem (mean)	16.3 ± 5.4	14.2 ± 5.4	14 ± 5.6
Brainstem (max)	40.7 ± 3.4	35.2** ± 5.5	34.0** ± 5.2
CTV coverage	98.4% ± 1.8	98.8% ± 0.8	98.8% ± 1.0
V105	4.7% ± 4.6	4.6% ± 3.6	3.2% ± 3.8
Conformity index	0.70 ± 0.08	0.73 ± 0.07	0.74 ± 0.06
Heterogeneity index	1.17 ± 0.34	1.10 ± 0.19	1.03 ± 0.24
Total MU	891 ± 91	925 ± 80	819** ± 70
Delivery time (min)	10.9 ± 1.3	11.3 ± 1.3	2.5** ± 0.1

* *P* < 0.05 compared to split field.

** *P* < 0.01 compared to split field. Doses for organ sites in Gy ± SD.

**FIG. 3:**
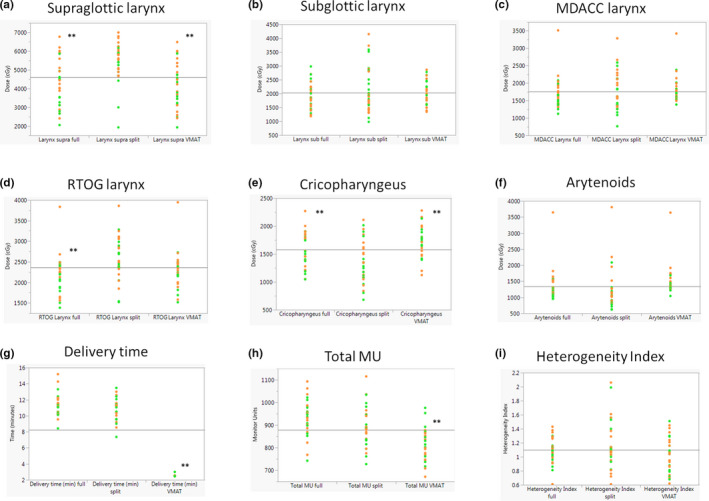
Comparisons between the three treatment techniques for (a) mean supraglottic dose, (b) mean subglottic dose, (c) mean dose to the MD Anderson Cancer Center‐defined larynx, (d) mean dose to RTOG 1016 defined larynx, (e) mean dose to the cricopharyngeus, (f) mean dose to the arytenoids, (g) total delivery time in minutes, (h) total MU required for each plan and (i) the heterogeneity index. Patients treated to 70 Gy are depicted with orange markers while those treated to 66 Gy are shown in green. **P < 0.05 referenced to the SF‐IMRT plans.

WF‐IMRT and VMAT, compared to SF‐IMRT, demonstrated significantly lower mean dose to the RTOG larynx (*P* < 0.01), supraglottic larynx (*P* < 0.01), esophagus (*P* < 0.01) and spinal cord (*P* < 0.01). By contrast, the cricopharyngeus mean dose was significantly lower with SF‐IMRT (13.7 Gy) (*P* < 0.05) compared to WF‐IMRT (16.0 Gy) and VMAT (17.8 Gy) (*P* < 0.05). The arytenoid mean dose trended lower for SF‐IMRT (11.9 ± 6.4 Gy) compared to VMAT plans (14.9 ± 4.5 Gy) (*P* = 0.07). There were no significant differences observed for the MDACC larynx and subglottic larynx among treatment techniques. Selected subsites are graphically represented in Fig. 3.

### 3.C Larynx and laryngeal substructure dose stratified by gross mid neck disease

Laryngeal and substructure dose after stratification by the presence or absence of gross nodal disease in the mid‐neck are shown in Table 3. The presence of gross mid‐neck disease significantly increased dose to the supraglottic larynx, RTOG larynx and esophagus, but did not impact dose to the MDACC larynx or arytenoids. The cricopharyngeus benefitted from SF‐IMRT in those with gross mid‐neck disease (*P* < 0.01) but not in the absence of mid‐neck disease. By contrast, the supraglottic and RTOG larynx benefitted most from non‐SF‐IMRT in those with gross mid‐neck disease. Differences in MDACC larynx dose by technique were not impacted by presence or absence of mid‐neck disease.

**TABLE 3:** Dose comparison stratified by the presence of mid‐lower neck disease.

	Level 3 neck disease absent			Level 3 neck disease present		
	Split field	WF‐IMRT	VMAT	Split field	WF‐IMRT	VMAT
Supraglottic larynx	56.5 ± 12.1	45.7 ± 14.4	43.6* ± 13.7	52.7 ± 9.2	38.0** ± 10.3	38.4** ± 9.6
Subglottic larynx	22.2 ± 8.5	17.5 ± 4.7	19.9 ± 5.0	20.3 ± 8.2	21.2 ± 4.1	20.9 ± 3.8
MDACC larynx	18.9 ± 6.2	17.4 ± 5.5	18.8 ± 4.9	16.6 ± 4.8	16.3 ± 2.7	17.2 ± 2.3
RTOG larynx	26.4 ± 5.8	22.5 ± 5.8	23.3 ± 5.7	25.2 ± 4.4	21.7* ± 2.9	22.5 ± 2.4
Cricopharyngeus	14.6 ± 4.1	14.8 ± 3.0	16.8 ± 3.1	12.7 ± 3.3	17.2** ± 2.9	18.9** ± 2.4
Arytenoids	12.6 ± 7.7	13.4 ± 6.3	15.1 ± 5.8	11 ± 4.6	13.5 ± 2.5	14.8* ± 2.5
Esophagus	35.4 ± 4.9	15.5** ± 4.8	16.0** ± 5.0	36.7 ± 4.7	21.1** ± 4.4	20.8** ± 4.5

* *P* < 0.05 compared to split field.

** *P* < 0.01 compared to split field. Doses in Gy ± SD.

### 3.D Treatment time and dosimetric indices

The VMAT plans had a significantly shorter treatment time (2.5 ± 0.1 min vs 10.9 ± 1.3 min vs 11.3 ± 1.3 min, *P* < 0.01) and required fewer monitor units (819 ± 70 vs 891 ± 91 vs 925 ± 80, *P* < 0.01) as compared with the SF‐IMRT and WF‐IMRT plans. There were no significant differences among techniques in terms of target coverage, dose conformity or the heterogeneity index. T‐ and N‐staging, primary involving the tonsil or tongue base, or prescribed dose, did not correlate with larynx dose or dosimetric indices.

## 4 DISCUSSION

Dysphagia is a significant treatment‐associated toxicity following head and neck radiotherapy. Mean laryngeal doses exceeding 40 Gy are associated with risk of aspiration and prolonged feeding tube dependence.^17^, ^18^ Despite the need to minimize unnecessary larynx dose, published data evaluating the optimal method for laryngeal sparing in OPSCC patients receiving IMRT have yielded mixed results.

As early adopters of IMRT for head and neck cancer treatment, we previously demonstrated a considerably lower mean MDACC larynx dose with SF‐IMRT (18.7 Gy), compared to WF‐IMRT (47 Gy) in 13 patients with early stage OPSCC (T1‐2, N0‐1).^13^ These results supported the use of SF‐IMRT to mitigate laryngeal dysfunction risk despite potential disadvantages such as match line dose uncertainties through gross disease and longer treatment times from the additional LAN fields.

In this update, we evaluated a homogeneous cohort of 30 patients with locally advanced OPSCC (excluding N3 and laryngeal involvement) treated to the bilateral neck. The MDACC larynx dose was similar across techniques (18.1 Gy SF‐IMRT, 16.7 Gy WF‐IMRT and 16.5 Gy VMAT). In all cases except one, a mean larynx dose < 29 Gy (MDACC) and <33 Gy (RTOG) was achieved irrespective of technique (Figs. 3(c) and 3(d)). Notably, excellent laryngeal sparing with a mean dose < 19 Gy (MDACC larynx) was achieved in 70% of patients in this study irrespective of technique. No differences were found between tonsil and tongue base primaries.

To better identify the optimal laryngeal dose‐volume, we evaluated two common CT‐based 3D larynx definitions. We defined the MDACC larynx volume based on the cranio‐caudal extent of the thyroid cartilage as previously reported in Dabaja et al, whereas the RTOG larynx was defined according to the RTOG 1016 protocol for HPV‐associated OPSCC and included the supraglottic and subglottic volumes. Across all techniques, the mean MDACC laryngeal dose (17.6 ± 4.6 Gy) was lower than the RTOG‐defined larynx (23.6 ± 4.7 Gy) (*P* < 0.05). This difference was attributed to the inclusion of the higher doses to the supraglottic structures included in the RTOG larynx volume. Whole‐field techniques did provide better sparing of the supraglottic volume compared to SF‐IMRT.

Several factors may explain the lower larynx dose in this study compared to our prior observations. In addition to a larger sample size and improvements in CT‐based contouring and IMRT treatment planning, this study used a non‐uniform PTV for whole‐field techniques such that PTV expansion into the larynx was restricted. In Dabaja et al, a 3‐mm uniform PTV was employed for WF‐IMRT with no restrictions into the laryngeal OAR. The lack of PTV expansion into the larynx volume reflects our current clinical practice which mirrors the historic LAN field in which there is no further expansion of the radiation field beyond the larynx block. In both the prior and current study, we did not correct for the differences in total dose to volumes below the match line between SF‐IMRT (50 Gy in 25 fractions) and whole‐field techniques, which used a simultaneous integrated boost (either 54 in 30 fractions or 57 Gy in 33 fractions). Thus, given no difference in larynx dose between the techniques, the biological dose to the larynx may actually be higher with SF‐IMRT than WF‐IMRT and VMAT.

**FIG. 4:**
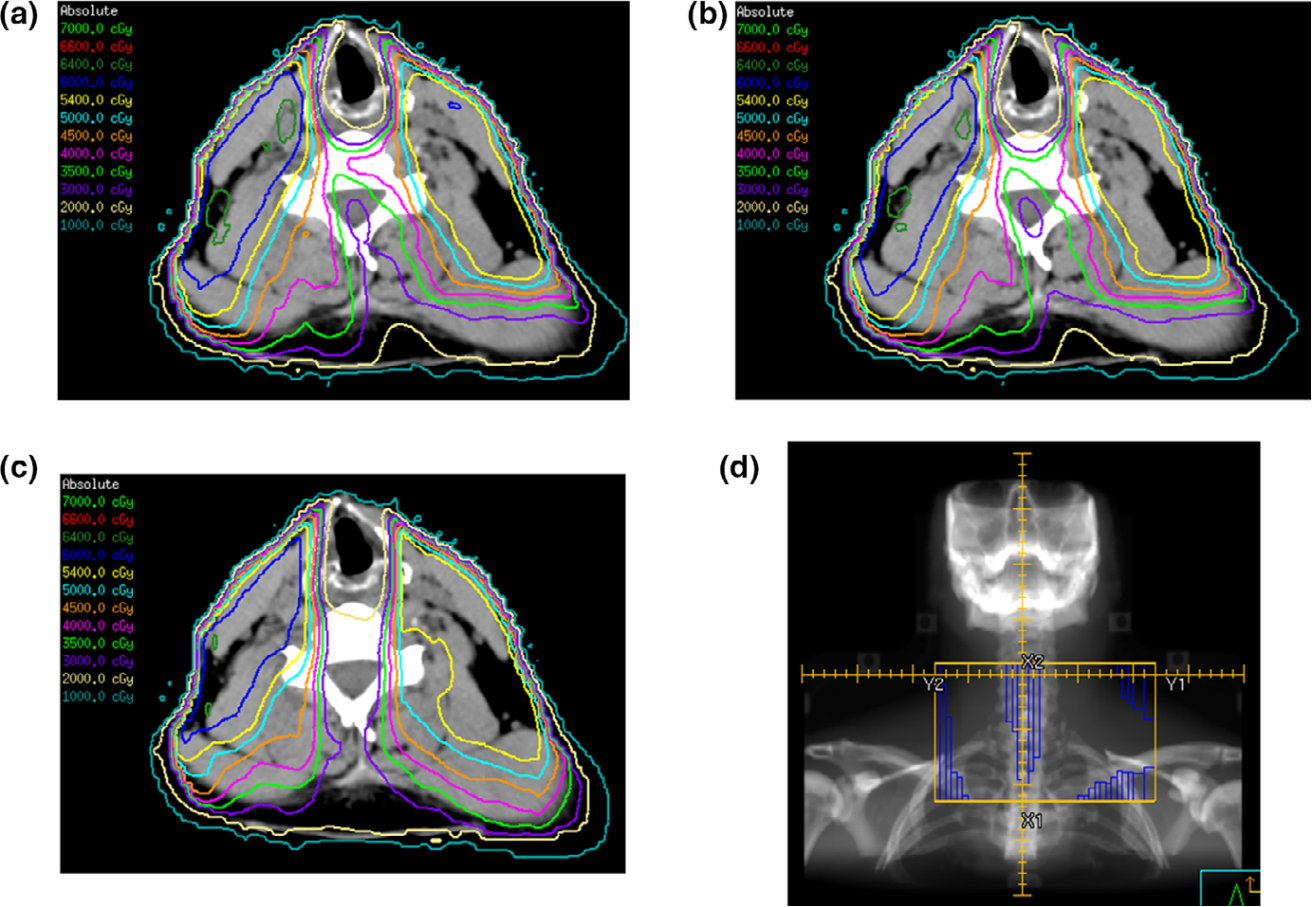
Optimization of the volumetric modulated arc therapy (VMAT) planning process. (a) Standard VMAT plan with evidence of high dose bowing into the region between the larynx and spinal cord (mean larynx dose = 25.6 Gy) (b) VMAT plan with addition of posterior larynx avoidance planning constraints (mean larynx dose = 24.6 Gy), (c) VMAT plan with vertical MLC optimization (mean larynx dose = 23.7 Gy) and (d) example demonstrating the vertical MLC orientation.

We also evaluated the impact of gross nodal disease in level 3 on the larynx dose. This appeared to have a higher impact on whole‐field approaches versus split‐field approaches. In particular, a higher cricopharyngeus dose correlated with the presence of gross disease in level 3 using WF‐IMRT and VMAT but not SF‐IMRT. Moreover, a significant portion of the region between the larynx and spinal cord, including the constrictor muscles, may receive a significantly higher unintended radiation dose that may not be reflected in the laryngeal volume with whole‐field techniques [see Fig. 4(a)]. To minimize leakage of unintended doses to this region, we recommend incorporating at a minimum a posterior laryngeal avoidance structure during treatment planning if the posterior constrictors are not included as OARs [Fig. 4(b)]. To further reduce dose to the larynx and constrictors with WF‐IMRT and VMAT, we are currently investigating the use of vertical MLC orientations in the low neck fields while also maintaining a high‐degree of target conformity [Figs. 4(c) and 4(d)]. Using this method, the vertical MLC field in WF‐IMRT and sagittal arc in VMAT can serve as a larynx block similar to the midline block used in SF‐IMRT plans.

Multiple studies with smaller cohorts have demonstrated correlations between radiotherapy dose to the larynx and constrictor muscles and risk of clinical dysphagia.^19^, ^20^, ^21^, ^22^ While our results demonstrate significant differences in dose to several organ‐at‐risk sub‐sites with the use of ST‐IMRT vs WF‐IMRT and VMAT planning, it remains unclear if the relative modest differences we observed would translate into a meaningful clinical benefit. Nevertheless, in comparison to our prior publication comparing SF‐IMRT with WF‐IMRT techniques, we no longer observe significantly worse mean laryngeal doses with modern whole field techniques thanks to intervening advances in treatment planning and delivery.

This study also highlights the need for consensus contouring guidelines regarding the larynx and sub‐sites for radiotherapy plan evaluation as well as for longitudinal impact of radiation dosing on long‐term swallowing function. Although the larynx is a known at‐risk organ for post‐treatment radiation‐induced dysphagia, there is a paucity of published guidelines for contouring of the larynx and laryngeal substructures and their and dose‐volume constraints for swallowing dysfunction.^23^ Our study has several findings that are noteworthy for clinical application. First, we demonstrate significant variations in achievable mean doses to various laryngeal substructures regardless of treatment planning technique, which urges the consideration and adoption of a standard larynx volume definition with correspondingly stricter dose constraints. The current quantitative analyses of normal tissue effects in the clinic (QUANTEC) guidelines recommend a maximum laryngeal dose of 66 Gy (for primary larynx tumors) or a mean dose < 44 Gy to coincide with a 20% or less chance of edema. Second, careful selection of treatment planning technique can aid in optimizing dose sparing of laryngeal substructures. In addition to avoiding potential disadvantages of a split‐field technique, both WF‐IMRT and VMAT can provide reductions in dose to the supraglottic larynx without compromising target coverage in appropriate patients. To further reduce laryngeal dose when using whole field techniques, a larynx avoidance OAR larger than the anatomic larynx volume can be created that extends posterior to the larynx to encompass the anterior portion of the vertebral body. Doing so minimizes dose invagination in between these structures where the pharyngeal constrictor muscles reside.

Finally, the obvious advantages with VMAT over static‐field IMRT are more efficient plans with fewer monitor units and shorter treatment times. According to an Australian study, the implications associated with VMAT was a 34% reduction in cost, with likely greater real economy savings when considering the cost for new construction and machines to treat the same number of patients as IMRT in the same time frame.^24^ In this study, VMAT was considered logistically and economically equivalent to 3D CRT with the dosimetric advantages of IMRT. Clinical advantages are improved dose conformity, more dose homogeneity and lower risk of intrafractional motion related to VMAT technique.^24^

Potential limitations of this study include the exclusion of oropharyngeal cancer patients with bulky nodal disease or primary tumors extending to the larynx and the lack of long term follow up data regarding dysphagia. Dosimetric indices for SF‐IMRT plans were based on actual treatment plans and it is conceivable that these plans could have been optimized further. Conversely, the WF‐plans were not used in treatment of patients and thus the final plans were “accepted” for this study when the planning objectives were achieved without further optimization beyond this.

In conclusion, modern IMRT techniques including SF‐IMRT, WF‐IMRT, and VMAT can provide excellent laryngeal sparing for patients with primary oropharynx cancer not involving the larynx who receive bilateral neck radiotherapy. There were no major clinical dosimetric differences with the exception of supraglottic larynx mean doses among IMRT techniques. VMAT was the most efficient treatment method requiring significantly fewer monitor units and considerably shorter treatment duration and thus lower risk of intrafraction movement. Achieving a larynx mean dose <20 Gy was feasible in a majority of cases and in nearly all cases, a mean larynx dose of <30 Gy was accomplished using all three techniques. VMAT should be considered the treatment of choice for OPSCC when appropriate as it provides equivalent or better dosing, a faster treatment time, and improved MU efficiency.

## CONFLICT OF INTEREST

Dr. Fuller receives academic‐industrial institutional grant funding from Elekta AB, and has received travel compensation from Elekta AB.
